# Trends in prevalence of selected opportunistic infections associated with HIV/AIDS in Uganda

**DOI:** 10.1186/s12879-015-0927-7

**Published:** 2015-04-17

**Authors:** John Rubaihayo, Nazarius M Tumwesigye, Joseph Konde-Lule

**Affiliations:** Department of Epidemiology and Biostatistics, School of Public Health, College of Health Sciences, Makerere University, Kampala, Uganda; Department of Public Health, School of Health Sciences, Mountains of the Moon University, Fort Portal, Uganda

## Abstract

**Background:**

After more than a decade of establishing and expanding access to highly active antiretroviral therapy (HAART), empirical evidence on its impact on trends of opportunistic infections (OIs) associated with the deadly human immunodeficiency virus (HIV) in resource poor settings is scarce. The primary objective of this study was to assess the effect of HAART coverage on trends of five most common OIs in Uganda.

**Methods:**

Observational data from January 2002 to December 2013 for 5972 HIV positive individuals attending the AIDS Support Organisation (TASO) HIV/AIDS care programme in Uganda were extracted and analysed. Trends were analysed using autoregressive moving average time series and mixed effects linear regression models adjusting for all available potential confounders.

**Results:**

A total of 204,871 monthly medical reports were retrieved and analysed. Majority of the participants were female (73%) with a median age of 32 years (inter-quartile range 26–39). Overall, significant decreasing mean annual prevalence trends were observed for Mycobacterium tuberculosis, herpes zoster, genital ulcer and oral candidiasis (p < 0.05, X^2^_trend_). Non-significant declining mean annual prevalence trend was observed for cryptococcal meningitis (p = 0.181, X^2^_trend_). The largest impact of HAART was observed in Oral candidiasis and TB whose average annual prevalence reduced by 61% and 43% respectively following the introduction of HAART. Monthly series for TB, Herpes zoster and genital ulcers differed significantly by age and clinic but only genital ulcer series differed significantly by sex (p < 0.05, kruskal wallis). After controlling for the effects of age, sex and clinic (fixed) and monthly clustering (random effect) in a mixed effects linear regression model, all the five OIs showed a significant monthly change in prevalence (p < 0.001).

**Conclusion:**

Overall, prevalence of most OIs declined especially after the introduction of HAART. However significant variations exist in the trends of different OIs in different geographical areas in Uganda. It is therefore important that site specific factors are properly identified to enable the development of targeted interventions.

## Background

Since the outbreak of HIV in 1981, an estimated 39 million people worldwide have died and about 35 million are living with the deadly virus with Sub-Saharan Africa suffering the greatest brunt of the epidemic [1]. Opportunistic infections (OIs) remain the single main cause of ill-health and death among HIV-infected patients [2-4]. Research shows that about 90% of HIV-related morbidity and mortality are caused by opportunistic infections compared to 7% due to opportunistic cancers and 3% due to other causes [5]. However, this may have changed since the introduction of HAART in mid-1990s in developed countries [6-10]. HAART is known for effective suppression of systemic HIV viral load and immune restoration thereby reducing the frequency of opportunistic infections, deferring morbidity and mortality hence improving survival among HIV infected individuals [7,11,12].

Several developing countries are slowly scaling up access to HAART, amidst scarcity of resources and uncertainty for a sustained lifelong provision of treatment to an ever increasing number of eligible HIV patients [1]. By end of 2013, about 13million HIV patients had access to HAART globally with 9.2million from middle and low income countries [13]. In resource poor settings, HIV positive individuals usually access care and treatment with marked immune suppression associated with a higher risk of OIs whose spectrum and frequencies may vary over time and in different countries or even within the same country [3,14]. OIs lower the quality of life of persons living with HIV/AIDS (PLHA), increases stigma and limits one’s ability to work and are usually associated with high medical care costs. Opportunistic infections therefore have greatly contributed to poverty among those infected and affected by HIV hence may be an impediment to the attainment of the millennium development goals (MDGs) on health and poverty eradication in resource poor settings.

Previous studies in developed countries show varied results on the effect of HAART on opportunistic infections over time and in different geographical areas [11,15-17]. For example a study in the USA that evaluated annual trends for 13 most common AIDS-defining opportunistic infections by examining medical records in more than 90 hospitals and clinics in 9 US cities before HAART (1991–96) showed decreasing trends in 5 OIs (PCP, esophageal candidiasis, tuberculosis, herpes simplex and cryptosporidiosis) and an increasing trend in recurrent pneumonia [11]. The trends in the time of onset, spectrum and frequency of infections was found to be unique for different OIs and varied by level of immune-suppression [11].

In another study in the USA, opportunistic infection rates varied considerably among US-born, Mexican-born and central American-born Latinos in the era of HAART. U.S.-born Latino women were more likely than Central American born Latino women to develop an OI (hazard ratio = 2.9, CI: 1.3, 6.5). In a Poisson regression analysis, U.S.-born Latino men and women combined were at greater risk of Kaposi’s sarcoma (RR 2.9, 95% CIs: 1.1, 7.6, p = 0.03) and yet for esophageal candidiasis, there was no evidence of a change in rate between the three communities [18].

Another study in the USA that reviewed the trends in the epidemiology of opportunistic fungal infections associated with HIV/AIDS reported an increasing trend in the incidence of fungal infections (*Aspergillus* sp) attributed to increasing resistance to anti-fungal treatment and recommended documentation of epidemiological trends to gain more insights into the effectiveness of treatment strategies [19]. A study in Spain that examined temporal trends in the incidence of opportunistic infections (OIs) associated with AIDS in the period 1989–1997 showed a significant decreasing trend in esophageal candidiasis, pulmonary and extra-pulmonary tuberculosis, and cerebral toxoplasmosis. However an increasing but non-significant trend of MAC incidence over time [20]. In Brazil, trends of OIs among HIV-infected adults (>12 years old) at a national level in the period between 1980 and 1999 declined significantly but not homogeneously by regions, risk groups, education and sex [21]. In Northeast and Central-West regions, they showed an increasing trend for TB and Toxoplasmosis. TB had higher incidence among those with lower education (>8 years) while PCP and KS had higher incidence among those with 8+ years of education, despite having similar trends of decline.

Few studies have examined the effect of HAART on trends of OIs in the African settings. One of these few studies examined the trends in incidence rates of TB in HIV-negative adults in South Africa before HAART and reported an increasing trend of TB incidence (test for trend *P* = 0.17) [22]. Given the scale and speed at which HAART roll-out is taking place in sub-Saharan Africa and in view of the policy changes in HAART access over time [23], it is important to evaluate the impact of these costly HIV interventions in resource poor settings. Uganda is among African countries with the highest burden of HIV/AIDS [24]. Currently there are an estimated 1.5 million people living with HIV/AIDS with about 60% in need of HAART [13]. HAART rollout began in 2004 as part of the Global HAART roll out strategy and the national target is to provide HAART to 80% of the population in need by 2015 [25]. However, the impact of the increasing coverage of HAART on trends of OIs in the country has previously not been examined due to lack of sufficient data. The primary objective of this study was to evaluate the impact of HAART coverage on trends of five most common OIs over time and in different geographical areas in Uganda.

## Methods

### Study design

Observational data for 5972 HIV positive individuals were obtained from the AIDS support organisation (TASO) in Uganda and analysed. The period of study was from January 2002 to December 2013 including 2 years before and 10 years after HAART introduction in Uganda. The OIs of interest were cryptococcosis, tuberculosis, oral candida, herpes zoster and genital ulcer. These were chosen on the basis of being the commonest OIs among HIV+ individuals in Uganda and are easily diagnosed. The review period was categorised into three mutually exclusive time periods that signify important milestones in the prevention and treatment of opportunistic infections associated with HIV/AIDS in Uganda. First period (2 years) was from January 2002 to December 2003 in which HIV+ individuals had no access to HAART. Second period (5 years) was from January 2004 to December 2008 denoted as “Early HAART” in which HAART access was limited to severely ill HIV+ individuals (CD4 count <200 cells/μl of blood) as per World Health organisation 2006 HAART access guidelines [26]. Third period (5 years) was from January 2009 to December 2013 denoted as “Late HAART” in which a lot of experience in HAART administration had been gained and HAART access policy changed to include all HIV+ individuals with CD4 cell count ≤350 cells/ μl of blood as per World Health organisation 2010 HAART access guidelines [27].

### Settings

The study was conducted in TASO, one of the oldest and largest HIV/AIDS care and treatment non-governmental organisation (NGO) in Uganda and sub-Saharan Africa [28]. TASO has 11 HIV clinics nationally recognised as centres of excellence with excellent laboratory practice and management supported by Centre for Disease Prevention and Control (CDC) [29]. With close to 3 decades of experience in HIV/AIDS care and treatment, TASO provided a good opportunity for assessing OI trends in real life programmatic settings in which HAART roll out is taking place in sub-Saharan Africa [30]. TASO ART programme started as part of the National ART roll-out programme in public health facilities in 2004. Being one of the largest ART providers in the country, TASO got support from different funders supporting ART programmes in sub-saharan Africa including the President’s Emergency Plan for AIDS Relief (PEPFAR) and the Global Fund to Fight AIDS, Tuberculosis and Malaria. Initially, access to HAART was based on the Ugandan ministry of Health and the World Health Organisation (WHO) 2006 guide lines i.e. WHO stage 3 or 4 illness or a CD4 cell count < 200 cells/μl for adults and adolescents and WHO stage III, advanced stage II or stage I with CD4 cell percentage less than 20% for those more than 18 months of age [26]. However, after 2010 access to HAART was expanded to include HIV patients with CD4 cell count ≤ 350 or WHO clinical stage3 or 4 irrespective of CD4 cell count as was recommended by Ugandan Ministry of Health and WHO [27,31]. Clients who are not eligible for HAART are usually offered cotrimoxazole or dapson prophylaxis. By end of 2013, a total of 91,218 were actively in care of which 58,051 clients (64%) were on HAART and 87,903 (96.4%) were on cotrimoxazole/dapson prophylaxis throughout the 11 HIV clinics across Uganda [29]. All services are free of charge including anti-retroviral drugs (ARVs) for those who are eligible [30]. Though TASO data system was not set up to answer any particular research question, but with assistance from Centre for Prevention and Disease Control (CDC) and other partners, TASO has been able to put up a large and up to date HIV/AIDS data base that can be used to inform and guide national policy on HIV/AIDS in Uganda [28].

### Sampling and sample size

Three TASO HIV clinics were purposively selected basing on volume and quality of data necessary for analyzing trends of most common OIs as well as less common OIs and geographical representation. The HIV clinics selected were TASO Mulago HIV clinic in central Uganda, TASO Mbarara HIV clinic in south-western Uganda and Tororo HIV clinic in Eastern Uganda. All clients irrespective of age who were in care at the three selected HIV clinics by 1^st^ January 2002 were included in the study.

### Data collection

Data on OIs were obtained from the TASO electronic data bases at the three HIV clinics and TASO headquarters in Kampala. The data were collected by TASO medical staff following an established protocol for all TASO HIV clinics. In brief, each client was expected to attend the clinic at least once a month. At each clinic visit, data per client was collected on a standardized medical form detailing the client’s demographic information, clinical condition, medical history, OI diagnosis, ART use and level of adherence, prophylaxis use, any other treatment given and side effects/toxicities if any. Diagnosis of OIs was based on the Ugandan ministry of health guidelines: Sputum tuberculin positive test or a chest radiography and/or GeneXpert positive test for TB; A CRAG positive test for Cryptococal meningitis; persistent creamy white curd-like plagues or red patches on the tongue, palate or lining of the mouth for oral candida; progressive and painful genital ulceration for genital ulcers and painful vesicular skin blisters with a dermatomal distribution for herpes zoster. All medical data were compiled and entered into the TASO electronic data base by TASO data administrator using EPIINFO vs3 in Access format. Monthly medical data for each participant covering the period January 2002 to December 2003 were extracted by the data administrator, delinked from overt identifiers and then handed over for analysis.

### Data management and analysis

Data management and analysis was done using STATA 12 (Stata Corp, Collage station, Texas, USA). Data were exported from epiinfo/access format to STATA 12 and cleaned. Data cleaning involved data reduction, deleting duplicates and missing data checks. To analyse trends, data on each OI was re-assembled and summarised by year and month. Then monthly and average annual prevalence for each OI were plotted. Monthly prevalence was calculated from the total number of clients recorded with an event in a month divided by the total number that attended the clinic in that month. Annual prevalence per OI was calculated from the mean of the monthly prevalences in a year.

To establish whether there is a trend, prevalence of each OI by month and calendar year were first tabulated and then a monthly time plot for prevalence of each OI was drawn. To filter out short-term fluctuations and random variation within monthly trends, we used the Box Jenkins moving average smoothing technique [32]. The moving average smoothing technique achieves this by replacing each element of the time series by *n* neighbouring elements, where n is the width of the smoothing window. We used a centred moving average including 3 observations before and 2 observations after the current observation inclusive, and then generated smoothed 144 monthly prevalence series per OI.

Annualized prevalence estimates were computed for each OI and *X*^2^ test for linear trend used to test for the significance of the trends. To measure the rate of change in monthly prevalence (b-coefficient) for each OI, we used mixed effects linear regression and modelled between monthly variability as a random effect. To test for the significance of the difference between trends, we used kruskal wallis test [33]. A scatter plot and histogram of the residuals were used to assess for assumption underlying linear regression (i.e. linearity, normal distribution and homogeneity of the variance). Monthly prevalence scatter plots that were non-linear (curvilinear) were first log-transformed before modelling the monthly rate of change in mixed effects linear regression.

### Ethical considerations

The study obtained ethical approval from Makerere University School of Public Health Higher Degrees Research and Ethics committee and the Uganda National Council for Science and Technology. Informed consent from study participants was not required as this was routinely collected operational data and the above ethical committees waived the need for consent. However, written consent was obtained from TASO for conducting study and publication of findings with any accompanying images.

## Results

### Baseline characteristics

A total of 204,871 monthly medical reports from a cohort of 5972 HIV positive patients were retrieved and analysed. 73% (4301) were female with a median age of 32 years (inter-quartile range 26–39). More than half of the study participants were rural poor peasants (56%) with majority having only primary level education (50%) and 30% were widowed (Table 1) . By 2004, only 9% of these had access to HAART but by 2013, all (100%) who were still in care were on HAART. The overall median age at ART initiation was 44 years (inter-quartile range 37–50) but was significantly lower for women at 43 (inter-quartile range 37–49) than that of men at 45 (inter-quartile range 40–52) (p = 0.001). The median CD4 cell count at the start of HAART was 128 cells (inter-quartile range 55–190) and the most commonly used HAART regimes were zidovudine plus lamivudine and nevirapive (ZDV + 3TC+ NVP) (44%), followed by stavudine, lamivudine and nevirapine (d4T + 3TC + NVP) (40%), and other regimen combinations that includes efavirenz (EFV), Tenofovir (TDF) and Lopinavir/ritonavir, etc. were rarely used (20%) (Table 2).

**Table 1:** **Baseline characteristics of the cohort at the start of the study, total and clinic-specific**

**Variable**	**Total cohort** ***n(%)***	**Tororo HIV clinic** ***n(%)***	**Mulago HIV clinic** ***n(%)***	**Mbarara HIV clinc** ***n(%)***
**Sex (n = 5,972)**				
**Female**	4,301(73)	1,071 (71)	1,368 (76)	1,862 (70)
**Male**	1,671 (27)	433 (29)	436 (24)	802 (30)
**Median age(IQR) (n = 5964)**	32 (26,39)	33 (28,40)	30(25,36)	32 (27,39)
**Occupation (n = 5031)**				
**paid employee**	627(12)	120(8)	232(18)	275(12)
**self employed**	1125(22)	271(18)	336(27)	518(23)
**peasant/unemployed**	2808(56)	932(62)	563(45)	1313(58)
**others**	471(9)	196(13)	127(10)	148(7)
**Education (n = 5,005)**				
**None**	1199(23)	414(27)	135(11)	650(29)
**Primary**	2521(50)	789(52)	623(50)	1106(49)
**Secondary**	1063(21)	286(19)	415(33)	364(16)
**Tertiary or above**	220(4)	22(1)	82(6.5)	116(5)
**Marital status (n = 5029)**				
**Single(never married)**	195(4)	24(2)	98(8)	73(3)
**Married**	2074(21)	690(45)	441(35)	943(42)
**Divorced**	678(13)	152(10)	324(26)	202(9)
**Widowed**	1504(30)	462(30)	236(19)	806(36)
**Others**	578(11)	191(13)	159(13)	228(10)

**Table 2:** **Mean number of study participants who were in care and those on ART each year**

**Year**	**Total number in care**	**Total number LFU/died n(%)**	**Total on ART n(%)**	**Tororo HIV clinic n(%)**		**Mulago HIV clinic n(%)**		**Mbarara HIV clinic n(%)**	
				In care	On ART	In care	On ART	In care	On ART
**2002**	5972	-	**-**	**-**	**-**	**-**	**-**	-	**-**
**2003**	4725	1247(−21)	**-**	**-**	**-**	**-**	**-**	-	**-**
**2004**	3896	829(−18)	371(9)	1181	-	1185	252(21)	1530	119(8)
**2005**	3551	345(−9)	837(23)	1261	61(5)	1157	343(30)	1133	433(38)
**2006**	2980	571(−16)	934(32)	972	71(7)	931	392(42)	1077	471(44)
**2007**	2401	579(−19)	1051(43)	863	84(10)	686	438(64)	852	529(62)
**2008**	2087	314(−13)	1135(54)	823	101(12)	581	466(80)	683	568(83)
**2009**	2127	42(+2)	1281(60)	887	176(20)	579	497(86)	661	608(92)
**2010**	2190	63(+3)	1693(77)	888	557(63)	570	514(90)	632	622(98)
**2011**	2002	188(−9)	1741(87)	689	577(84)	558	524(94)	655	640(98)
**2012**	1724	278(−14)	1724(100)	578	578(100)	515	515(100)	631	631(100)
**2013**	1772	48(+3)	1772(100)	592	592(100)	533	533(100)	647	647(100)

LFU = Lost to follow up.

Pre-ART (2002–2003) retention was 79% (4725/5972) after 24 months of follow up. Post HAART (2004–2013) mean annual retention was 90% (2237/2473) with an average of 10% (236/2473) loss to follow up per year. In sensitivity analysis, the female gender of those who remained in care did not differ significantly from those who attended but did not return (*X*^2^ = 0.475, P = 0.491). The median age only differed by 1 year (*X*^2^ = 19, P < 0.001), with those who remained in care having median age of 32 yrs (inter-quartile rage 27–39) while those who were lost to follow up had a median age of 31 (inter-quartile rage 26–38). So we assumed cases that remained in care were a representative sample of all the cases and that missing clinic visits happened randomly.

### Prevalence and trends of OIs

**Figure 1:**
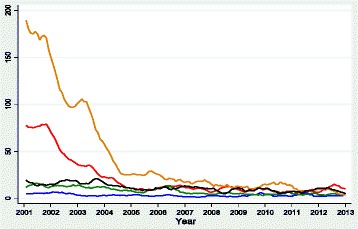
Monthly prevalence trend for oral candida (orange line), Mycobacterium tuberculosis (redline), genital ulcer (black line), herpes zoster (green line) and cryptococcal meningitis (blue line) expressed as a proportion of HIV-positive persons diagnosed with a particular OI out of the total number who turned up for care per month for the period January 2002 to December 2013.

**Figure 2:**
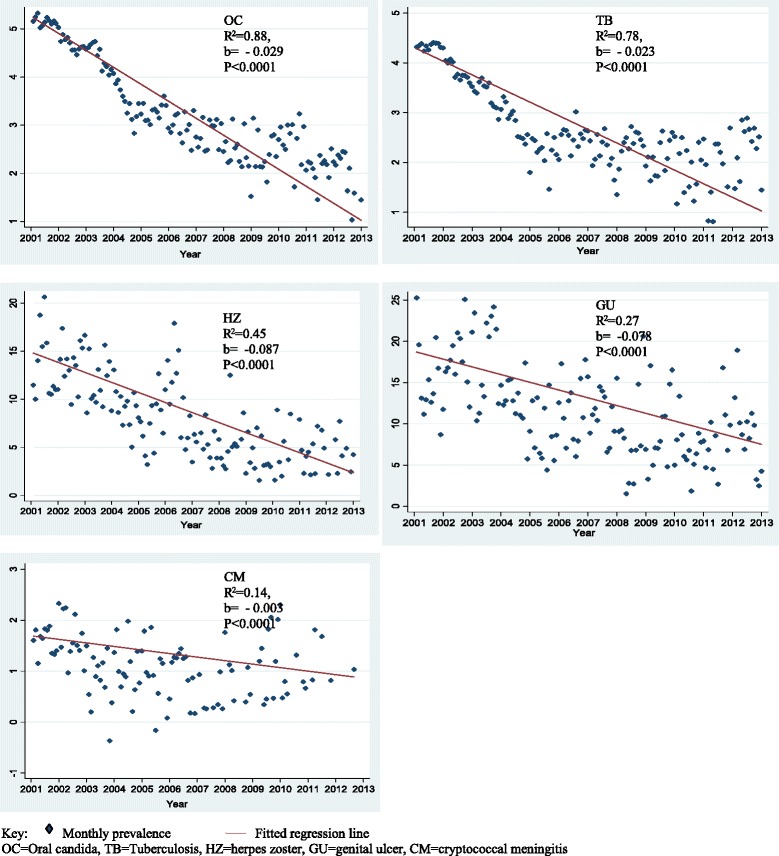
Scatter plot and fitted regression line for monthly prevalence over time (in months) for each OI expressed as a proportion of HIV-positive persons diagnosed with a particular OI out of the total number who turned up for care per month for the period January 2002 to December 2013.

**Figure 3:**
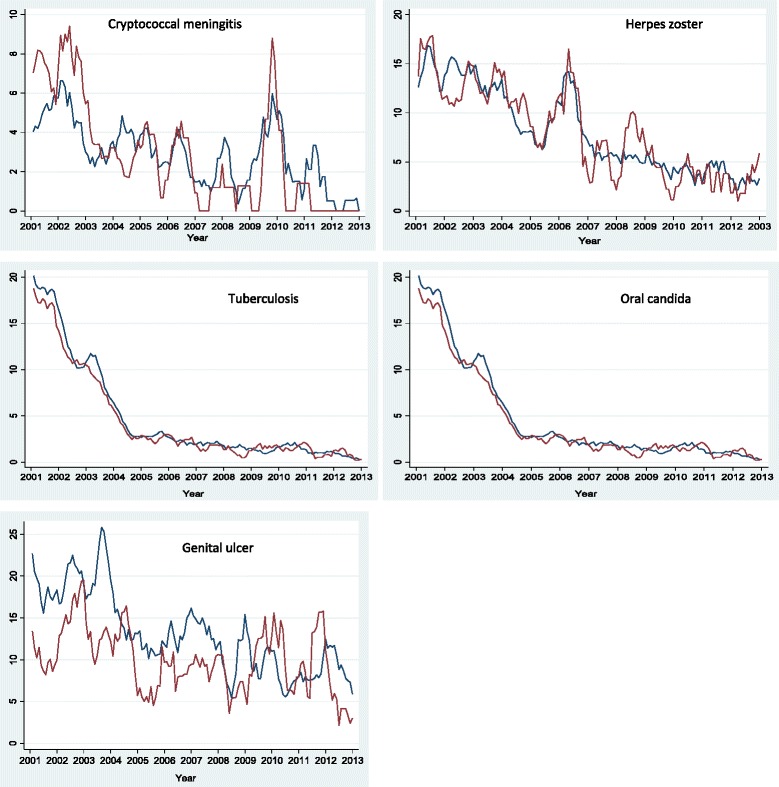
Monthly prevalence trend for each OI by sex: female (blue line), male (maroon line); expressed as a proportion of HIV-positive persons diagnosed with a particular OI out of the total number who turned up for care per month for the period January 2002 to December 2013.

**Figure 4:**
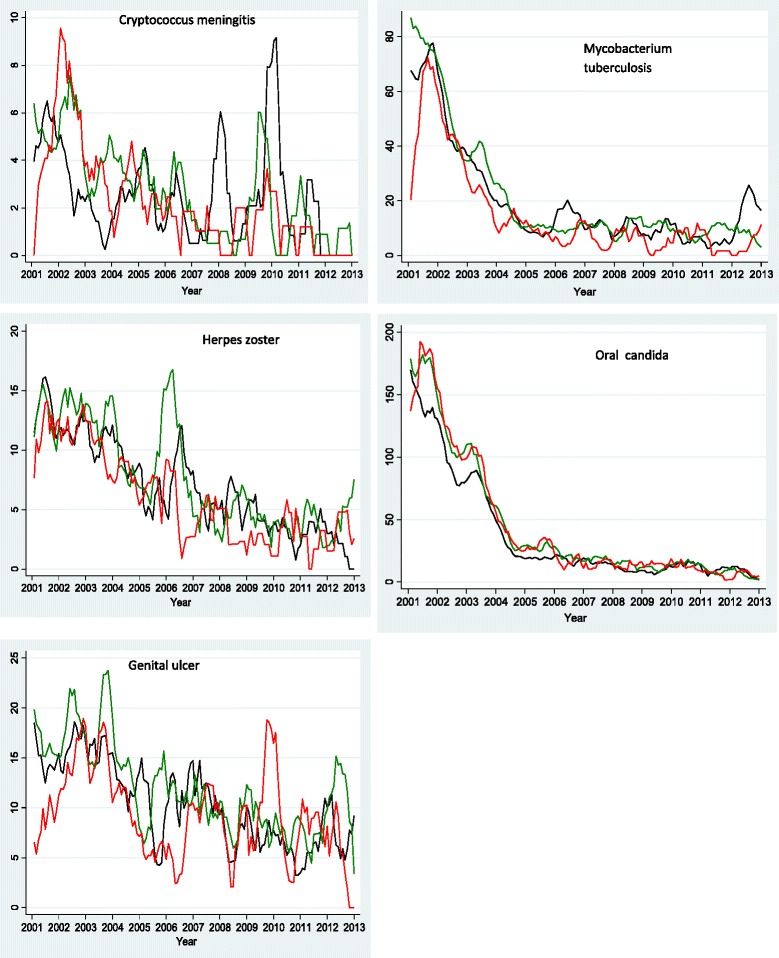
Monthly prevalence trend for each OI by age: <30 yrs (blackline), 30-39 yrs (greenline) & 40 yrs and above (redline); expressed as a proportion of HIV-positive persons diagnosed with a particular OI out of the total number who turned up for care per month for the period January 2002 to December 2013.

Overall monthly trends for each OI are shown in Figure 1. Best-fit regression lines, R^2^ and p-values for each OI are shown in Figure 2. Baseline characteristics at the commencement of HAART are shown in Table 2. Numbers of study participants accessing HAART over time are shown in Table 3. Average annual prevalence by each OI and statistical tests for trend are shown in Table 4. Average monthly changes in OI prevalence over time after controlling for age, sex and clinic as fixed effects and monthly clustering as a random effect are shown in Table 5. Figures 2, 3 and 4 show monthly prevalence trend comparisons by sex, age and HIV clinic. Genital ulcer and Herpes zoster monthly prevalence decreased linearly with time, whereas TB and oral candida monthly prevalence trends exhibited a curvilinear decrease with time, decreasing rapidly between 0-48months and thereafter levelled off in the following months.

**Table 3:** **Background characteristics of the study participants at commencement of HAART, total and sex segregated**

**Characteristic**	**Total (N = 1741)**	**Female (N = 1413)**	**Male (N = 328)**	p-value*
**Age in years, median (IQR)**	44(37,50)	43(37,49)	45(40,52)	0.001
**WHO stage III & IV, n (%)**	1113(63.9)	929(65.7)	184(56.1)	0.121
**CD4 cells/μl, median (IQR) (2005–6)**	128(55,190)	144(65,191)	49(39,92)	0.074
**ART regime (2005–2013), n (%)**				
**d4T + 3TC+ NVP**	693(40)	638(45)	136(41)	0.233
**ZDV +3TC + NPV**	774(44)	562(40)	131(40)	
**other**	274(16)	213(15)	61(19)	

Key: *Chi^2^-Test; IQR = Interquartile range; ART = Antiretroviral therapy; d4T = Stavudine; 3TC = Lamivudine, NVP = Nevirapine; ZDV = Zidovudine, WHO = World Health Organisation.

**Table 4:** **Mean annual OI prevalence (per 1000) in a cohort of HIV positive individuals before and after HAART in Uganda**

	**Before HAART**		**Early HAART**					**Late HAART**					
**OI**	**2002**	**2003**	**2004**	**2005**	**2006**	**2007**	**2008**	**2009**	**2010**	***2011***	***2012***	***2013***	***X*** ^***2***^_***trend***_ ***(p-value)***
**Cryptococcal meningitis**	5.5	5.4	2.5	3.3	2.7	2.7	1.6	1.2	4.0	1.3	1.3	0.23	1.79 (0.1807)
**% change**	-	0	−54	+32	−18	0	−41	−25	+233	−67	0	−82	
**Mycobacterium tuberculosis**	76.9	45.5	29.3	16.2	9.6	13.2	9.0	10.9	9.1	7.4	6.6	11.0	157.38 (<0.0001)
**% change**	-	−41	−36	−45	−41	+37	−32	+21	−16	−19	−11	+67	
**Herpes zoster**	13.4	13.9	11.5	9.1	7.6	10.0	5.1	5.0	3.8	3.7	3.7	3.3	13.67 (0.0002)
**% change**	-	+4	−14	−25	11	+37	−54	2	−24	−20	0	−11	
**Oral candida**	172.7	106.4	83.1	32.3	26.5	20.0	17.0	12.0	12.6	15.4	8.7	6.7	458.20 (<0.0001)
**% change**	-	−38.7	−20	−62	−16	−26	−10	−33	+8	−23	−37	−20	
**Genital ulcer(HSV-2)**	15.1	18.2	17.6	12.3	9.3	12.0	11.2	8.1	9.2	7.2	7.7	8.9	8.8 *(*0.0029*)*
**% change**	-	+21	3.3	−33	−17	+20	−8	27	+25	−20	−12.5	+43	

Key: HAART = Highly active antiretroviral therapy; % = percent; HSV-2 = Herpes simplex virus type2.

**Table 5:** **Mixed effects linear regression analysis of monthly rate of change in the prevalence of each OI (per 1000) adjusted for fixed effects (age, sex and clinic) and random effects (monthly clustering)**

	**Cryptococcal meningitis** ^**1**^	**Mycobacterium tuberculosis** ^**2**^	**Herpes zoster**	**Oral candida** ^**3**^	**Genital ulcer**
	**β(95% CI)**	**β (95% CI)**	**β (95% CI)**	**β (95% CI)**	**β (95% CI)**
	**[p-value]**	**[p-value]**	**[p-value]**	**[p-value]**	**[p-value]**
**Predictor variable (Fixed effect)**					
Time*	−0.003 (−0.004 to −0.002) [<0.001]	−0.023 (−0.023 to −0.022) [<0.001]	−0.087 (−0.09 to −0.08) [<0.001]	- 0.0296 − 0.0298 to −0.0293) [<0.001]	−0.078 (−0.08 to −0.07) [<0.001]
**Sex**					
**Female**	1	1	1	1	1
**Male**	0 .0058 (−0.042 to 0.054) [0.813]	0.035 (0.010 to 0.059) [0.004]	0.15 (−0.55 to 0.24) [0.449]	−0.008 (−0.021 to 0.004) [0.197]	0.083 (−0.45 to 0.61) [0.758]
**Age**					
**<30 yrs**	1	1	1	1	1
**30**–**39**	−0.022 (−0.073 to 0.028) [0.387]	−0.021 (−0.046 to 0.004) [0.096]	0.11 (−0.29 to 0.51) [0.588]	0.0002 (−0.012 to 0.013) [0.980]	0.004 (−0.48 to 0.47) [0.986]
**40+**	−0.041 (−0.10 to 0.020) [0.187]	- 0.035 (−0.066 to −0.003) [0.032]	−0.05 (−0.54 to 0.44) [0.841]	0.007 (−0.007 to 0.021) [0.341]	0.49 (−0.11 to 1.09) [0.110]
**HIV clinic**					
**Tororo**	1	1	1	1	1
**Mulago**	−0.022 (−0.073 to 0.028) [0.387]	−0.089 (−0.12 to −0.062) [<0.001]	0.22 (−0.26 to 0.70) [0.373]	−0.02 (−0.042 to −0.002) [0.032]	0.25 (−0.84 to 0.34) [0.414]
**Mbarara**	−0.091 (−0.15 to −0.035) [0.001]	−0.12 (−0.15 to −0.088) [<0.001]	0.10 (−0.35to .55) [0.666]	−0.020 (−0.038 to −0.002) [0.028]	0.45 (−1.04to 0.13) [0.130]
**Constant**	0.77	4.39	14.7	5.3	18.6
**β**_**0**_	(0.70 to 0.84) [<0.001]	(4.35 to 4.42) [<0.001]	(14.0 to 15.5) [<0.001]	(5.2 to 5.3) [<0.001]	(17.5 to 19.8) [<0.001]
**Predictor variable (Random effect)**					
**Month**					
**δ** ^**2**^_**μ**_	0.006	0.001	0.94	0.002	2.8
**(95% CI)**	(0.0023 to 0.017)	(0.0005 to 0.004	(0.39 to2.26)	(0.001 to 0.005)	(1.2 to 6.5)
**[SE]**	[0.003]	[0.0006]	[0.42]	[0.001]	[1.20]
**δ** ^**2**^_**ε**_	0.041	0.11	0.33	0.060	17.2
**(95% CI)**	(0.036 to 0.048)	(0.10 to 0.11)	(7.6 to 9.1)	(0.058 to 0.062)	(16.0 to 18.5)
**[SE]**	[0.003]	[0.0026]	[0.368]	[0.001]	[0.62]

Key: *Time =144 months, β = beta coefficient, SE = standard error, CI = confidence interval, δ^2^_ε_ = Residual variance.

δ^2^_μ_ = random effect monthly variance.

^1^Natural log monthly prevalence (per 1000) of Cryptococcal meningitis.

^2^Natural log monthly prevalence (per 1000) of Mycobacterium tuberculosis.

^3^Natural log monthly prevalence (per 1000) of Oral candida.

### Mycobacterium tuberculosis

**Figure 5:**
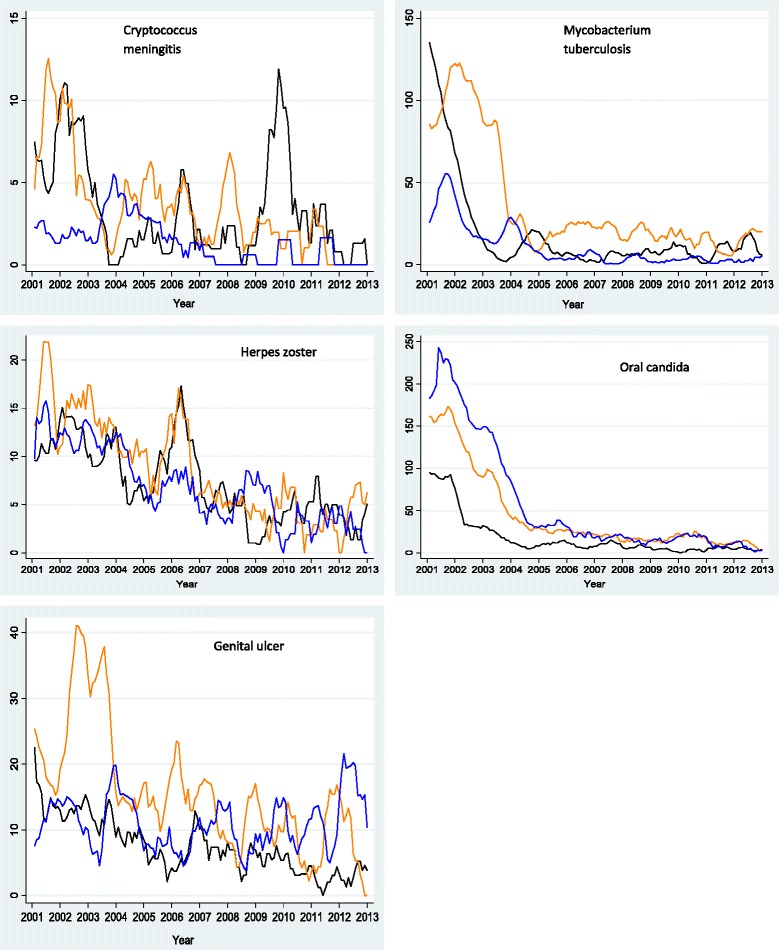
Monthly prevalence trend for each OI by HIV clinic: Mulago HIV clinic (orange line), Tororo HIV clinic (blackline), and Mbarara HIV clinic (blue line); expressed as a proportion of HIV-positive persons diagnosed with a particular OI out of the total number who turned up for care per month for the period January 2002 to December 2013.

TB monthly prevalence was curvilinear with a steep declining trend between 2003 and 2005 and thereafter dismal change in prevalence was observed (Figure 1). The mean annual prevalence dropped from 76.9/1000 persons at risk in 2002 to 6.6/1000 persons at risk in 2012 but slighly increased to 11.0/1000 persons at risk in 2013 (p < 0.0001, X^2^_trend_) (Table 4). The largest reduction occurred in 2005 (52%), but thereafter there was dismal change in prevalence (Table 4). TB monthly prevalence trends were not different by sex (*X*^2^ = 0.19, p = 0.66, kruskal wallis) (Figure 3) but differed significantly by age (*X*^2^ = 18.5, p = 0.0001, kruskal wallis) (Figure 4) and HIV clinic (*X*^2^ = 94.7,p = 0.0001, kruskal wallis) (Figure 5). After adjusting for age, sex and clinic as fixed effects and monthly clustering as a random effect in a mixed effects linear regression model, average monthly prevalence declined at a rate of 2% per month (p < 0.05). However the rate of decline differed significantly by age, sex and clinic (p < 0.05) (Table 5).

### Oral candida

Oral candida monthly prevalence also showed a curvilinear trend with a steep declining trend between 2003 and 2005 and thereafter dismal change in prevalence was observed (Figure 1). The mean annual prevalence dropped from 173/1000 persons at risk in 2002 to 6.7/1000 persons at risk in 2013 (p < 0.0001, X^2^_trend_) (Table 4). The largest reduction in mean annual prevalence occurred in 2005 (61%) (Table 4). The monthly Oral candida prevalence trends were not significantly different by sex (*X*^2^ = 0.6, p = 0.44, kruskal wallis) (Figure 2) and age (*X*^2^ = 1.7,p = 0.42, kruskal wallis) (Figure 3) but differed significantly by clinic (Figure 4) with Mbarara HIV clinic (south western region) showing the highest prevalence trend, followed by Mulago HIV clinic (central region) and then Tororo HIV clinic (eastern region) (*X*^2^ = 63.2, p = 0.0001, kruskal wallis). After adjusting for age, sex and clinic as fixed effects and monthly clustering as a random effect in a mixed effects linear regression model, average monthly prevalence declined at a rate of 3% per month (p < 0.001) (Table 5). However the rate of decline did not differ significantly by age or sex (p > 0.05) but differed significantly by clinic (p < 0.05) (Table 5)

### Herpes zoster

Herpes zoster monthly prevalence showed a consistent declining trend (Figure 1). The mean annual prevalence reduced from 13.4/1000 persons at risk in 2002 to 3.3/1000 persons at risk in 2013 (p = 0.0002, X^2^_trend_) (Table 4). Largest reduction in mean annual prevalence was observed in 2008 (53.4%) (Table 4). However, Herpes zoster monthly trends were not significantly different by sex (*X*^2^ = 0.08, p = 0.77, kruskal wallis) and clinic (*X*^2^ = 2.4, p = 0.29, kruskal wallis) but differed significantly by age (*X*^2^ = 7.2,p = 0.027, kruskal wallis). After adjusting for age, sex and clinic as fixed effects and monthly clustering as a random effect in a mixed effects linear regression model, monthly prevalence declined significantly at an average rate of 0.087 per month (p < 0.001). However this rate of decline did not differ significantly by age, sex and clinic (p > 0.05) (Table 5).

### Genital ulcer

The prevalence of genital ulcer was relatively lower when comapred to Oral candida and TB. Monthly prevalence slightly but significantly reduced over the observation period (Figure 1). The mean annual prevalence dropped from 15.1/1000 persons at risk in 2002 to 8.9/1000 persons at risk in 2013 (p = 0.0029, X^2^_trend_) (Table 4). Its monthly prevalence series were significantly different by sex with women having a higher prevalence trend than men (*X*^2^ = 14.8, p = 0.0001, kruskal wallis) (Figure 3). The monthly series were also significantly different by age with HIV positive clients aged between 30-39 yrs having a relatively higher prevalence compared to those aged below 30 years and those aged 40 years and above (*X*^2^ = 10.6, p = 0.005, kruskal wallis) (Figure 4). The monthly series also differed significantly by HIV clinic with Mulago HIV clinic (central region) showing the highest prevalence trend, followed by Mbarara HIV clinic (south western region) and then lastly Tororo HIV clinic (eastern region) (*X*^2^ = 45.3, p = 0.0001) (Figure 5). After adjusting for age, sex and clinic as fixed effects and monthly clustering as a random effect, monthly prevalence declined significantly at an average rate of 0.078 per month. However the rate of decline did not differ significantly by age or sex or clinic (p > 0.05) (Table 5).

### Crypotococcal meningitis

Crypotococcal meningitis prevalence was relatively the lowest in this cohort and showed a non-significant trend (Figure 1). The prevalence decreased between 2003 and 2004 but showed intermittent decreasing and increasing trends between 2005 and 2013 with a peak increase observed in 2010. The mean annual prevalence reduced from 5.5/1000 persons at risk in 2002 to 1.2/1000 persons at risk in 2009, then increased to 4.0/1000 persons at risk in 2010 and thereafter declined to 0.23/1000 persons at risk in 2013 (p = 0.181, X^2^_trend_). The monthly series were not significantly different by sex (*X*^2^ = 3.48, p = 0.062, kruskal wallis) and age (*X*^2^ = 3.7, p = 0.15, kruskal wallis) but were significantly different by HIV clinic (*X*^2^ = 15.2, p = 0.0005, Kruskal Wallis). After adjusting for age, sex and clinic as fixed effects and monthly clustering as a random effect, monthly prevalence declined significantly at an average rate of 0.3% per month. However the rate of decline did not differ significantly by age or sex or clinic (p > 0.05) (Table 5)

## Discussion

On average globally, TB prevalence among HIV+ individuals slightly reduced from around 25% in 2007 to 21% in 2010 [34]. In the current study, we have observed a significant declining trend in mean annual prevalence of Mycobacterium tuberculosis. This is consistent with other previous studies, for example, a study in the USA that evaluated annual trends for 13 most common AIDS-defining opportunistic infections by examining medical records in more than 90 hospitals and clinics in 9 US cities before HAART (1991–96) and showed decreasing trends in tuberculosis [11]. Another study in Italy that examined temporal trends in the incidence of opportunistic infections (OIs) associated with AIDS in the period 1989–1997 showed significant decreasing trends in both pulmonary and extra-pulmonary tuberculosis [20]. However, differs from a cohort study in west London who investigated the incidence of 12 most frequent AIDS-defining illnesses in the pre-HAART and post-HAART time periods and found no significant reduction in the incidence of TB and other OIs but significant decrease in the incidence of PCP, Kaposis sarcoma and cryptosporidiosis [35].

Few studies though have examined the trends in prevalence of TB among HIV+ individuals in low income settings. One study in South Africa examined the trends in incidence rates of TB before HAART (1991–2000) reported a non-significant increasing trend of TB incidence (*P* = 0.17, X^2^_trend_) [22]. Another study in Malawi that examined trends in TB associated with HIV-infection between 1988 to 2001 showed incidence of active TB first increased up to 1990 and thereafter started to decline [36]. Uganda is among the 22 high-burden TB countries in the world [37] and previous studies in Uganda show that over 80% of the HIV-related morbidity and 30% of the HIV related death were due to TB [4,38]. TB co-infection with HIV has been associated with poor HAART prognostic outcomes [39]. A prospective cohort study that assessed the effect of HAART on TB incidence in Eastern Uganda showed TB reduced from 7.2% at baseline to 5.5% after 1.4 yrs of follow up [38]. Our findings are consistent with these previous findings and provide additional evidence of a significant HAART effect on TB prevalence in resource poor settings. However, the slight increase observed in 2013 implies that TB may never be completely eliminated since it is endemic in these settings and was even there before the advent of the HIV epidemic in Uganda.

Herpes zoster showed a significant declining trend though the effect of HAART seems to be relatively less compared to other OIs. Our findings are consistent with findings from a study in the USA that evaluated the effect of HAART on incidence trends of Herpes zoster from 1987 to 2011 and reported a significant reducing trend in annual incidence of Herpes zoster [40]. However our findings deviates from another study that followed HIV- infected patients from 1985 to 2003 in France found no significant difference in the incidence rate for Herpes zoster over time [41]. Another study in the USA reported an increasing trend in the prevalence of clinical Herpes zoster in the period from 1945 to 2008 [42]. Our findings provide additional evidence that increasing coverage of HAART is having a significant effect on Herpes zoster even in resource poor settings. However this effect on prevalence could also be attributed to increasing availability of other potent antiviral drugs like acyclovir.

Oral candida showed very sharp decline of 61% in 2005 probably because of the introduction of HAART in 2005 and increased availability of more potent systemic antifungal drugs like fluconazole. This is consistent with recent study findings in Spain which examined trends of candidiasis among HIV+ children (<15 years) for the period 1997–2008 and found significant decline in candidaisis diagnoses in this time period attributed to the effect of HAART . However, another study in the USA that reviewed the trends in the epidemiology of opportunistic fungal infections associated with HIV/AIDS showed a significant increasing trend in the incidence of fungal infections (*Aspergillus* sp) attributed to increasing resistance to anti-fungal treatment and recommended documentation of epidemiological trends to gain more insights into the effectiveness of treatment strategies [19]. Though a significant declining trend was observed, the OI has not been completely eliminated probably because it is highly endemic in Uganda or there could also be drug resistance which requires further investigations.

Genital ulcers probably caused by Herpes simplex virus type2 have previously been shown to be common among HIV positive patients [43-45]. A study in France found about 18% of the HIV infected adults were co-infected with herpes simplex virus type2 [46]. Herpes simplex virus-2 infection can be latent in the normal human body but become reactivated when the immune system is severely compromised [47]. In Uganda, a rural population study found the rate ratio for HSV-2 incidence was 3.69 in HIV-positive cases with genital ulcers compared to HIV-negative persons after adjusting for age and sex [43]. The study also found the prevalence of HSV-2 was much higher in women (71.5%) than in men (36.6%). Our study shows prevalence of genital ulcers declined slightly over time and the trends differed significantly by sex. A study in India reported an increasing prevalence trend of genital herpes between 2000 to 2004 but prevalence was reported higher in women compared to men [48]. Similar findings were also reported in another study in India [49]. Our findings provides additional evidence that increased availability of HAART and effective treatment particularly acyclovir could be responsible for the observed decline in the prevalence of genital ulcers in Uganda.

In the current study Cryptococcal meningitis showed a non-significant declining trend with a 50% reduction in 2004 probably due to the introduction of fluconazole around this time and highly active antiretroviral therapy in 2005 [50]. The overall declining trend is consistent with other previous studies in developed countries which also reported declining trends in the era of HAART. A study in Brazil [21] examined temporal trends in incidence of opportunistic infections (OI) among HIV-infected adults (>12 years old) at a national level for the period in 1980–1999. The study showed a declining trend for all OI including Cryptococcal meningitis among adult AIDS cases particularly after the introduction of HAART in 1996. In the USA, a population based surveillance for cryptococcosis conducted between 1992 and 2000 reported a declining trend in the incidence of cryptococcosis among HIV+ individuals attributed to widespread use of effective antiretroviral drugs [51]. Another study [19] that reviewed trends in epidemiology of opportunistic fungal infections showed that increasing use of effective antifungal prescriptions has reduced the frequency of invasive mycoses including cryptococcosis among HIV+ individuals in the US. Our study adduces more evidence that cryptococcosis is reducing among HIV+ individuals over time with increasing access to HAART and fluconazole in Uganda. However the slight increase in 2010 could be attributed to improvement in diagnosis following the introduction of the lateral flow cryptococcal Antigen (CrAg) rapid test strips [50,52]

The study also shows that complete elimination of opportunistic infections may not be possible partly because they are endemic and also as a consequence of gaps in timely access to treatment, poor adherence to HAART, inadequate staff and laboratory equipment, poverty and high patient attrition [53]. We also observed a mean annual attrition rate of 10% in the era of HAART whose cause we can only speculate. Previous studies have shown that despite the best efforts by care providers and donors to provide free lifelong ARVs, HIV patients would still drop out of programme care or withdraw from treatment for various reasons [54-57]. Sydney Rosen and colleagues in their systematic review on prevalence of loss to follow up among patients on ART, reported an average 40% prevalence of loss to follow up [56]. Some studies have also shown retention at 24 month in most ART programmes in Sub-Saharan Africa to be between 60-80% [56,58]. In Uganda, ART attrition rates after 12 months have been reported to reach up to 30% [53,59] while generally, among ART patients who are subject to attrition, i.e. who are lost to follow-up (LTFU), it is estimated that 30-60% are dead [53,59,60].

Lastly, missed clinic visits are inevitable over a lifelong HIV/AIDS care programme. However, our understanding of why people drop out of care and how to address this problem remains limited [61]. Norma Ware and colleagues conducted a large qualitative study among patients in HIV treatment programs in sub-Saharan Africa to investigate reasons for missed clinic visits and reported both intentional and unintentional reasons including cost of transport to the clinic, stigma and dissatisfaction with care, competing demands (both economic and social), finding an alternative source of care and discouragement due to perceived harsh treatment by care providers [57]. However, these factors are likely to vary from one geographical setting to another. Hence, it is crucial to correctly identify these factors so as to improve ART program retention and success in resource poor settings.

### Limitations

This study had a number of limitations. First, data analysed was limited to what was available in the TASO electronic data base and so certain variables whose data was not captured could not be analysed. Being a retrospective cohort, there was no information available on the clients who never returned for care. So we did not have information on the connection to care or survival status of clients who were lost to follow up. However we assumed that these simply relocated to another clinic for care or died as had been reported in other previous studies [54,55,59,62,63]. The TASO clients whose medical records were analysed may have not been representative of all HIV+ individuals in Uganda which means generalisability could be limited but still this does not compromise the evidence we have adduced since most if not all beneficiaries of public health care programmes in Uganda are generally the same. There may also have been a possibility that some OIs went away undiagnosed or were not recorded during clinic visits as was reported in a previous study conducted in Uganda [64]. Though we believe this was minimized by selected OIs which were easy to diagnose and of great interest in the country given that the study by Kiragga et al. [64] reported that the level of under reporting was far less for TB and Cryptococal meningitis compared to other less important OIs. From sensitivity analysis, we established that cases who remained in care were not significantly different from those that were lost to follow up and so we conservatively assumed that cases who remained in care were a representative sample of all the cases in the original cohort and that missing clinic visits happened randomly. Even with these limitations, there is sufficient evidence that increasing HAART availability in resource poor settings is having a meaningful impact on prevalence of opportunistic infections.

## Conclusions and recommendations

Overall, the data provides emperical evidence that the disease burden due to OIs has significantly reduced in the 12 years of follow up. The decrease in trends of individual OIs could be attribute to the increased access to HAART and other preventive prescriptions such as cotrimoxazole prophylaxis and effective therapeutic drugs over time. The study further shows that some opportunistic infections were still common though at reduced prevalence in spite of availability of effective treatment including HAART. This is probably because they are endemic or this could be a sign of resistance/treatment failure that needs to be further investigated. The differences in trends between OIs and geographical areas were likely to be due to differences in the level of exposure to infectious agents, social-economic status, drug resistance, immunity, nutrition, etc. Future research should explore the association of these factors with trends of individual OIs and knowledge of these factors could help in the design of taregeted interventions. The effect attributable to HAART alone or Cotrimoxazole prophylaxis and other therapeutic drugs was not established as this would require a comparison cohort which is exclusively on HAART alone which was not available. We recommend that future studies should examine this aspect. We also recommend that information on those lost to programme care should be routinely collected in order to have insight on the causes for drop out which will ultemately help to improve ART programmes and inform policy decisions on improvement of treatment outcomes in resource poor settings.
